# Procyanidin B2 Protects Aged Oocytes Against Meiotic Defects Through Cortical Tension Modulation

**DOI:** 10.3389/fvets.2022.795050

**Published:** 2022-04-08

**Authors:** Qingrui Zhuan, Jun Li, Guizhen Zhou, Xingzhu Du, Hongyu Liu, Yunpeng Hou, Pengcheng Wan, Xiangwei Fu

**Affiliations:** ^1^Key Laboratory of Animal Genetics, Breeding and Reproduction of the Ministry of Agriculture and Rural Affairs, National Engineering Laboratory for Animal Breeding, Beijing Key Laboratory for Animal Genetic Improvement, College of Animal Science and Technology, China Agricultural University, Beijing, China; ^2^Department of Reproductive Medicine, Reproductive Medical Center, The First Hospital of Hebei Medical University, Shijiazhuang, China; ^3^State Key Laboratories of Agrobiotechnology, College of Biological Sciences, China Agricultural University, Beijing, China; ^4^State Key Laboratory of Sheep Genetic Improvement and Healthy Breeding, Institute of Animal Husbandry and Veterinary Sciences, Xinjiang Academy of Agricultural and Reclamation Sciences, Shihhotze, China

**Keywords:** PCB2, reproductive aging, oocyte, cortical tension, meiotic maturation

## Abstract

Defects in meiotic process are the main factors responsible for the decreased developmental competence in aged oocytes. Our recent research indicated that natural antioxidant procyanidin B2 (PCB2) promoted maturation progress in oocytes from diabetic mice. However, the effect of PCB2 on aging-induced chromosome abnormalities and the underlying mechanism have not been explored. Here, we found that PCB2 recovered aging-caused developmental arrest during meiotic maturation, germinal vesicle breakdown (GVBD) rate was significantly higher in aged oocytes treated with PCB2 (*P* < 0.05). Furthermore, we discovered that cortical mechanics were altered during aging process, cortical tension-related proteins were aberrantly expressed in aged oocytes (*P* < 0.001). PCB2 supplementation efficaciously antagonized aging-induced decreased cortical tension (*P* < 0.001). Moreover, PCB2 restored spindle morphology (*P* < 0.01), maintained proper chromosome alignment (*P* < 0.05), and dramatically reduced reactive oxygen species (ROS) level (*P* < 0.05) in aged oocytes. Collectively, our results reveal that PCB2 supplementation is a feasible approach to protect oocytes from reproductive aging, contributing to the improvement of oocytes quality.

## Introduction

There is a global tendency that women delay conception until late 30's, by which time the chance of pregnancy is compromised as the reproductive capacity in women declines beyond their mid-30's (1). Reproductive aging deteriorates oocyte quality (2). It is known that maternal aging is associated with meiotic defects, and in addition to this, increased vulnerability of aged oocytes to reactive oxygen species (3) leads to mitochondrial dysfunction, since mitochondria are the most significant targets of oxidative stress (1, 4).

Cortical tension and stiffness experience dynamic changes through meiotic maturation and fertilization progression to facilitate and/or direct cellular remodeling in the mammalian oocyte (5). This cortex remodeling is part of the creation of cellular asymmetry and mediates the progression of the prophase I, germinal vesicle-intact (GVI) oocyte to the MII stage (6). The biochemical and structural features of the cortex are regulated by actin assembly, non-muscle myosin-II expression, and Ezrin/Radixin/Moesin (ERM) protein activity (5). A previous study indicated that abnormal cortical mechanics and myosin-II activity were associated with post-ovulatory aging (7), but changes in cortical tension during reproductive aging remain unclear.

Recently, antioxidants such as melatonin and resveratrol have received increasing attention in the development of therapeutic strategies against oocytes quality deterioration caused by reproductive aging (8, 9). Procyanidins, a group of plant polyphenols with powerful anti-oxidative properties, have been found effective in treating metabolic and inflammatory diseases (10, 11). Dimer procyanidin B2 [4,8′-BI- [4,8′-BI- [(+)-epicatechin]] (PCB2) is a member of oligomeric anthocyanins precursors, which can improve oocyte maturation and subsequent embryo development in diabetic mice (12, 13). In alcoholic liver disease model, dimer procyanidincan reduce hepatic lipid disposition and ROS over production, thereby activate hepatic autophagy to eliminate lipid droplets and damaged mitochondria (14). Furthermore, procyanidins also play a role in alleviation endoplasmic reticulum stress and metabolic disorders associated with endothelial dysfunction (15). However, the effect of PCB2 on the aged oocytes under oxidative stress and the mechanism underlying are not determined yet.

In light of this, the goal of the present study was to investigate the effect of reproductive aging on cortical tension in oocytes, and elucidate the role and the mechanism underlying PCB2 treatment in protecting oocytes from aging-caused quality declines.

## Materials and Methods

### Animals and Housing

All studies were performed using 8-week-old and 42–45-week-old CD-1^®^ (ICR) female mice (Vital River Laboratory Animal Technology Co., Ltd. Beijing, China). Mice were housed in ventilated cages on a 12 h light/12 h dark cycle (lights on from 08: 00 to 20: 00) under controlled temperature (22 ± 2°C) with freely available food and water. The mice were allowed to adapt to conditions for 7 days before the initiation of experiments. In this experiment, 42–45-week-old female mice nearly at the end of their reproductive lifespan were used as a natural aging model.

### Chemicals and Antibodies

All chemicals and drugs were purchased from Sigma (St. Louis, MO, USA) unless otherwise indicated. The anti-pERM antibody (#3726), anti-pMRLC antibody (#3675), and anti-rabbit IgG (H+L), F(ab')2 Fragment (Alexa Fluor^®^ 594 Conjugate) secondary antibody (#8889) were purchased from Cell Signaling Technology (Cell Signaling, USA). The anti-alpha Tubulin antibody (62204) was purchased from Thermo Fisher (Thermo Fisher Scientific, USA). The Fluorescein (FITC)–conjugated Affinipure Goat Anti-Rabbit IgG (H+L) secondary antibody (SA00003-2) was purchased from Proteintech (Proteintech Group, Inc.).

### Experimental Design

8-week-old young mouse oocytes were regarded as young group. 42–45-week-old aged mouse oocytes were randomly assigned to aged and PCB2-supplemented groups. Procyanidin B2 (PCB2) was dissolved in DMSO and diluted to a final concentration of 5 μg/mL with M16 or M2 medium, respectively. The youth group served as the control and received no treatment. The *in vitro* matured oocytes were randomly divided into three groups as follows: (1) young group: oocytes obtained from 8-week-old mice matured *in vitro*; (2) aged group: oocytes obtained from 42 to 45-week-old mice matured *in vitro*; (3) aged+PCB2 group: oocytes obtained from 42 to 45-week-old mice matured *in vitro* and treated with 5 μg/mL PCB2.

### Oocyte Collection

The mice were sacrificed by cervical dislocation 46–48 h after intraperitoneal injection of 10 IU pregnant mare serum gonadotropin (PMSG, Ningbo Hormone Product Co. Ningbo, Zhejiang Province, China). Fully-grown GV oocytes were collected by removing cumulus cells in a drop of M2 medium supplemented with dbcAMP (100 ng/mL) through repeatedly pipetting. Fully-grown GV oocytes were cultured in M16 medium under mineral oil at 37°C in 5% CO_2_ incubator.

### Immunofluorescence Staining (IF) and Confocal Microscopy

Oocytes were fixed with 4% (w/v) paraformaldehyde (PFA) for 40 min at room temperature, followed by permeabilization with 0.5% Triton X-100 at room temperature for 1h. After being blocked in 3% BSA for 1h at room temperature, oocytes/embryos were incubated with different primary antibodies (anti-pERM, 1:600; anti-pMRLC, 1:300; anti-α-tubulin, 1:8000; anti-β-tubulin, 1:100) overnight at 4°C. The oocytes were further incubated with FITC–conjugated Affinipure Goat Anti-Rabbit IgG (H+L) or Alexa Fluor 594-conjugated goat anti-rabbit antibody for 1 h at room temperature. Finally, all oocytes were stained with 4′,6-diamidino-2-phenylindole (DAPI) for 5 min at room temperature, then oocytes were mounted on glass slides and the fluorescent images were taken with a laser scanning confocal microscopy (A1 Cell Imaging System; Nikon) under the same staining procedure and confocal microscopy parameters. Mean fluorescence intensity per unit area within the region of interest was used to quantify the fluorescence intensity of each oocyte.

### Intracellular ROS Level Assay

Denuded oocytes were added to the medium which contains 1 mmol/L 2′, 7′-dichlorodihydrofluoresceindiacetate (DCFHDA) for measuring ROS at 37°C in 5% CO_2_ for 20 min. Then oocytes were washed by M2 three times. The fluorescence was examined under an epifluorescence microscope with a filter at 460-nm excitation for ROS (IX73; Olympus). The fluorescence of each oocyte was analyzed by EZ-C1Free-Viewer (Nikon).

### Statistical Analysis

In all experiments, data were analyzed using SPSS software v.21.0 (SPSS Inc., Chicago, IL, USA). Student's *t*-test was performed for statistical analysis. For abnormal spindle and chromosome alignment, chi-square test was performed for statistical analysis. Unless otherwise stated, ^*^ = *P* < 0.05, ^**^ = *P* < 0.01, ^***^ = *P* < 0.001, ns = non-significant difference (*P* > 0.05).

## Results

### PCB2 Promotes Meiotic Resumption in Aged Oocytes

**Figure 1:**
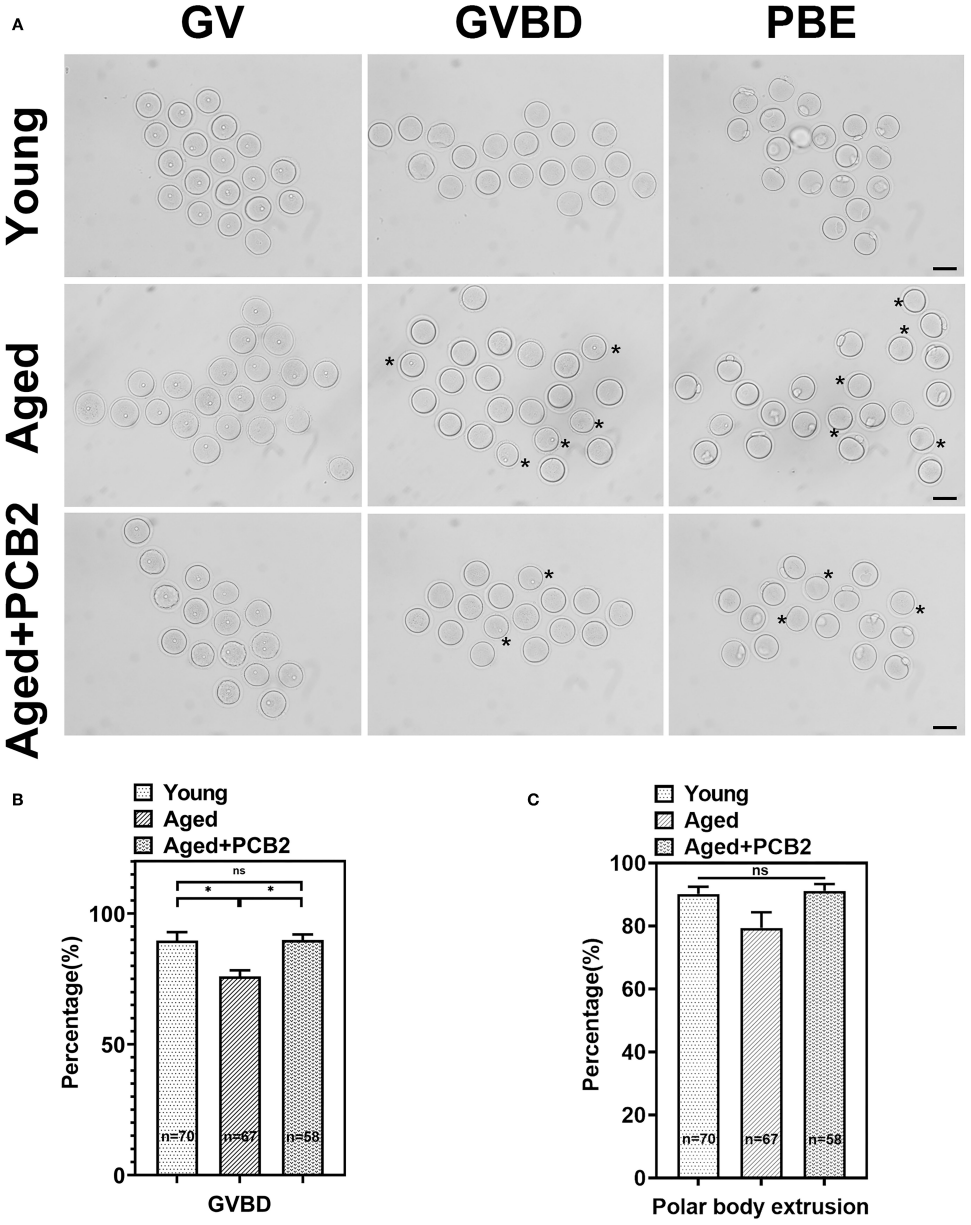
PCB2 supplementation can improve germinal vesicle breakdown (GVBD) rate of aged oocytes. **(A)** Oocytes were cultured *in vitro* with or without PCB2 supplementation to analyze GVBD and polar body extrusion (PBE) rates. Scale bar, 50 μm. **(B,C)** The GVBD and PBE rates of oocytes were recorded in different groups. Data are presented as mean percentage (mean ± SEM) of at least three independent experiments. *P < 0.05.

Meiotic resumption during *in vitro* maturation progress was examined. As shown in Figure 1A, a large majority of oocytes in the young group underwent germinal vesicle breakdown (GVBD) stage, then developed to the metaphase II (MII) stage with the extrusion of first polar body. However, oocytes in the aged group exhibited decreased GVBD rate at the same developmental time points (young: 89.67 ± 3.28%, *n* = 70; aged: 75.96 ± 2.33%, *n* = 67, *P* < 0.05). To determine the protective effect of PCB2 on meiotic recovery, fully-grown GV oocytes were cultured in M16 medium supplemented with 5 μg/mL PCB2. The quantitative analysis indicated that the oocytes treated with PCB2 exhibited significantly increased GVBD rate compared with the aged group (aged: 75.96 ± 2.33%, *n* = 67; aged+PCB2: 89.87 ± 2.13%, *n* = 58, *P* < 0.05, Figure 1B). However, after 12 h culture, there was no significant difference in the occurrence of polar body extrusion (PBE) among different groups (Figure 1C).

### PCB2 Recovers Aging-Related Sharp Decrease in pERM Expression

**Figure 2:**
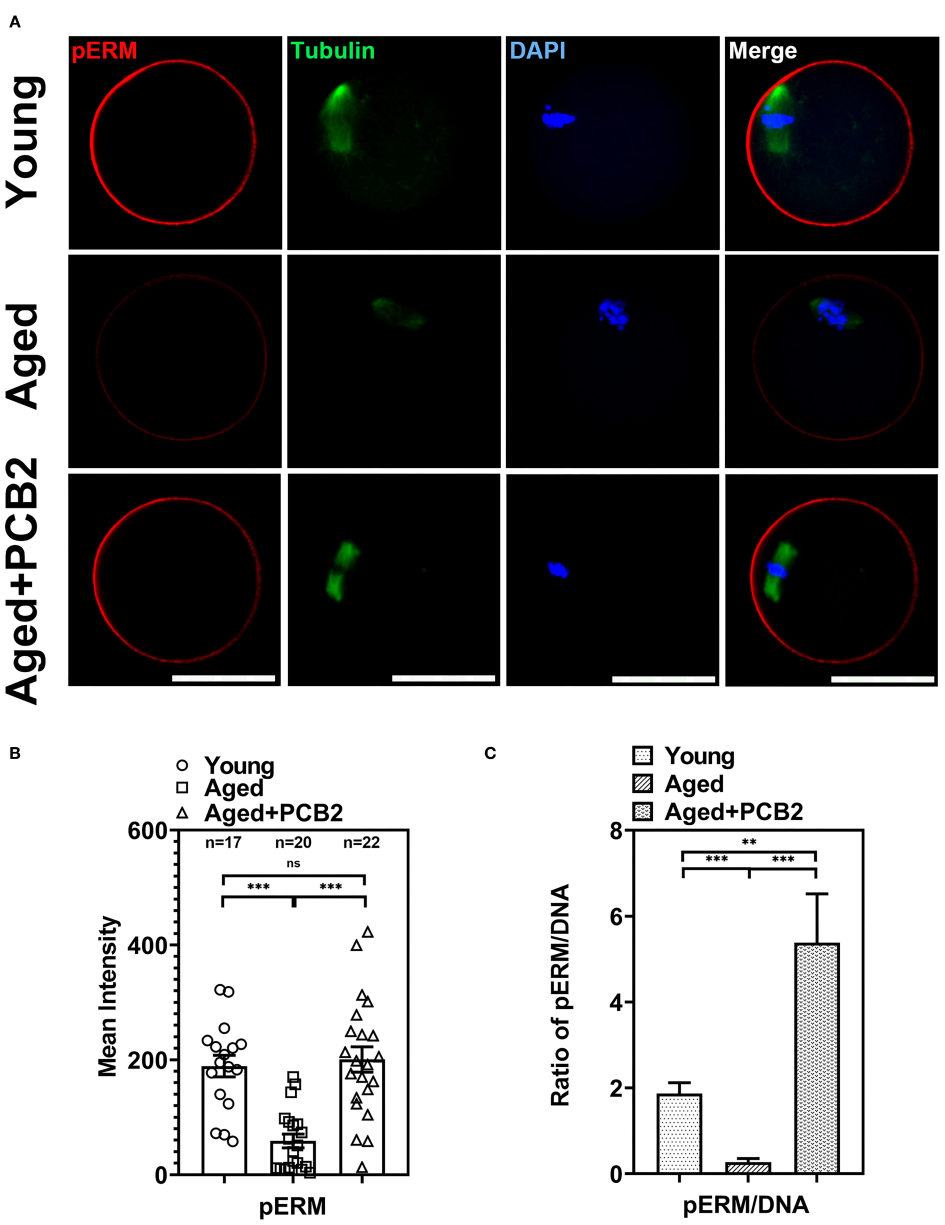
Effects of PCB2 on pERM expression and spindle formation during *in vitro* culture of aged oocytes. **(A)** IF staining of *in vitro* matured oocytes for pERM and tubulin. DNA was counterstained with DAPI (blue). Scale bar, 50 μm. **(B)** Fluorescence intensity of pERM signals was recorded in different groups. **(C)** The ratio of pERM to DNA fluorescence. Data are presented as mean percentage (mean ± SEM) of at least three independent experiments. **P < 0.01, ***P < 0.001. ns, non-significant difference, *P* > 0.05.

Previous study shown that post-ovulatory aged MII-stage oocytes exhibited abnormal cortical tension (7), which drives us to investigate whether reproductive aging can affect the cortical tension in MII-stage oocytes. We, therefore, conducted immunostaining assay to evaluate the expression of active phospo-ERMs (pERM), which play a crucial role in regulating cortical tension (Figure 2A). As shown in Figure 2B, quantitative analysis indicated that the fluorescence intensity of pERM was significantly reduced in aged oocytes (young: 189.14 ± 18.86, *n* = 17; aged: 43.02 ± 11.24 pixels, *n* = 16, *P* < 0.001), while PCB2 treatment rescued this abnormal phenomenon (aged: 43.02 ± 11.24, *n* = 16; aged+PCB2: 150.58 ± 28.28 pixels, *n* = 12, *P* < 0.001). In addition, DNA (DAPI staining) was used as internal reference to normalize the immunofluorescent staining results, and the ratio of pERM to DNA fluorescence further validated that PCB2 significantly increased pERM level in aged oocytes (Figure 2C).

### PCB2 Restores Abnormal Cytoplasmic Distribution of pMRLC in Aged Oocytes

**Figure 3:**
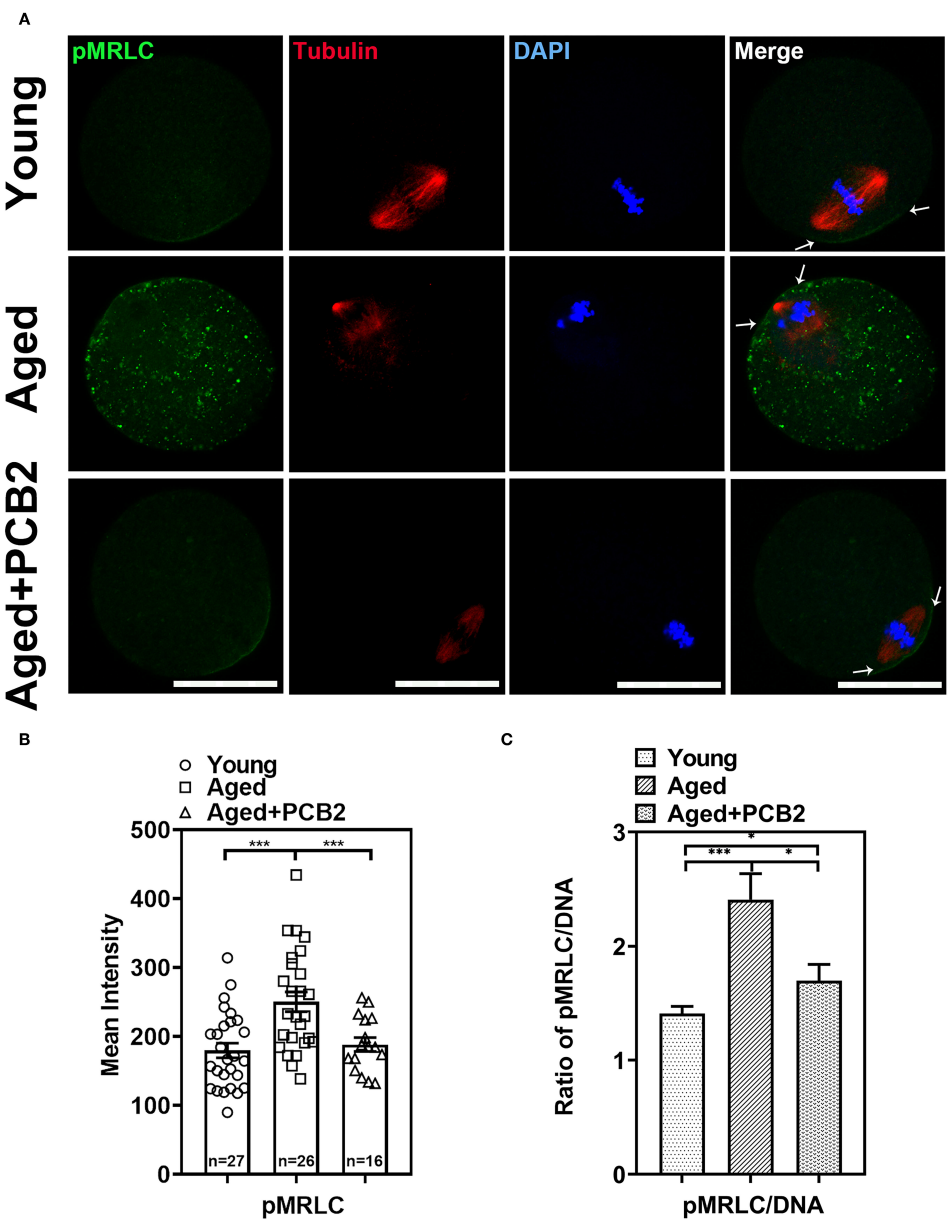
Effects of PCB2 on pMRLC expression and spindle formation during *in vitro* culture of aged oocytes. **(A)** IF staining of *in vitro* matured oocytes for pMRLC and tubulin. DNA was counterstained with DAPI (blue). Scale bar, 50 μm. **(B)** Fluorescence intensity of pMRLC signals was recorded in different groups. **(C)** The ratio of pMRLC to DNA fluorescence. Data are presented as mean percentage (mean ± SEM) of at least three independent experiments. *P < 0.05, ***P < 0.001. ns, non-significant difference, *P* > 0.05.

In addition to ERMs, non-muscle myosin-II is also a key factor regulating the cortical tension in oocytes (5). Reduced level of the active form of the myosin-II regulatory light chain [phosphorylated MRLC, pMRLC] was found in post-ovulatory oocytes (7). In the present study, normal localization of pMRLC in the amicrovillar domain (the region overlying the spindle, indicated with arrow in Figure 3A) was observed in most young oocytes. However, the amicrovillar domain distribution of pMRLC was disturbed in aged oocytes, and exhibited clustered distribution in the cytoplasm (Figure 3A). As expected, PCB2 treatment rescued this abnormal distribution. As shown in Figure 3B, the corresponding quantitative data showed a significantly increased pMRLC intensity in the cytoplasm of aged oocytes (young: 179.81 ± 10.67, *n* = 27; aged: 263.44 ± 15.54 pixels, *n* = 18, *P* < 0.001) and PCB2 rescued this phenomenon (aged: 263.44 ± 15.54, *n* = 18; aged+PCB2: 165.84 ± 23.20 pixels, *n* = 11, *P* < 0.001). Moreover, the ratio of pMRLC to DNA fluorescence further verified the above results (Figure 3C).

### PCB2 Maintains Normal Spindle Assembly and Protects Aged Oocytes From Chromosome Defects

**Figure 4:**
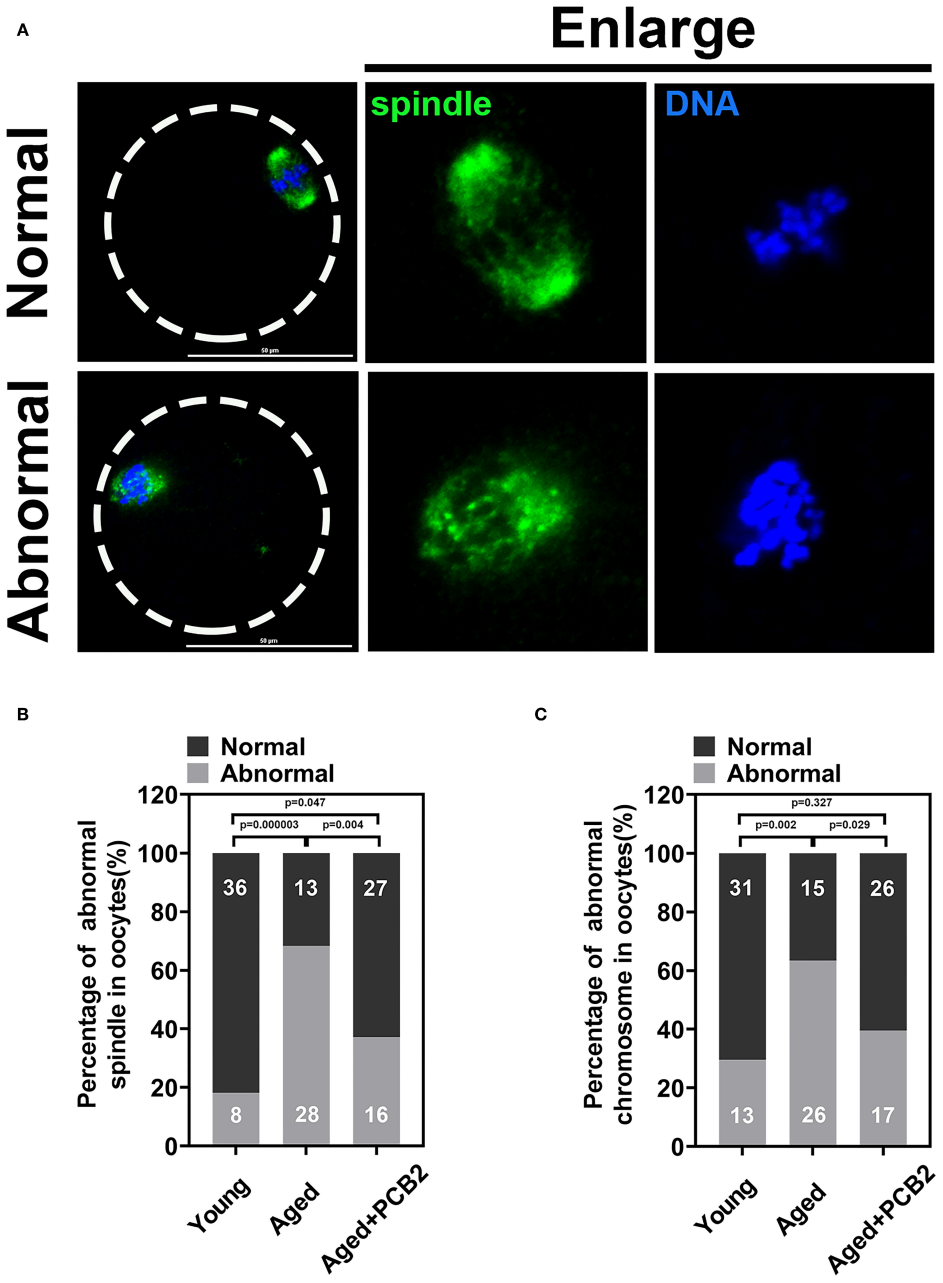
PCB2 alleviates abnormal spindle formation and misalignment of chromosome in aged oocytes. **(A)** Representative images of normal and abnormal spindle formation as well as chromosome alignment. Scale bar, 50 μm. **(B)** Quantification of normal and aberrant spindles was recorded in different groups. **(C)** Quantification of normal and misalignment chromosome was recorded in different groups. Chi-square test was performed for statistical analysis.

Normal spindle assembly is prerequisite for proper chromosome segregation. To delineate age-related changes in spindle architecture and chromosome aberrations, oocytes were immunostained with anti-α-tubulin antibody to observe the spindle morphology and counterstained with DAPI to analyze the chromosome alignment. As shown in Figure 4A, normal spindle displayed a typical barrel-shaped apparatus. Meanwhile, a well-aligned chromosome on the equatorial plate next to the cortical region was also fond of the oocyte membrane. However, disruptions in spindle organization and chromosomes alignment were observed in aged oocytes. PCB2 treatment not only rescued disorganized spindle assembly, but also recovered the misaligned chromosomes. As shown in Figure 4B, the corresponding quantitative data confirmed the protective role of PCB2 on spindle formation in aged oocytes (*P* < 0.01). Moreover, the quantitative data showed that PCB2 supplementation also rescued the chromosome abnormalities (*P* < 0.05, Figure 4C) in aged oocytes treated with PCB2.

### PCB2 Supplementation Attenuates ROS Levels in Aged Oocytes

**Figure 5:**
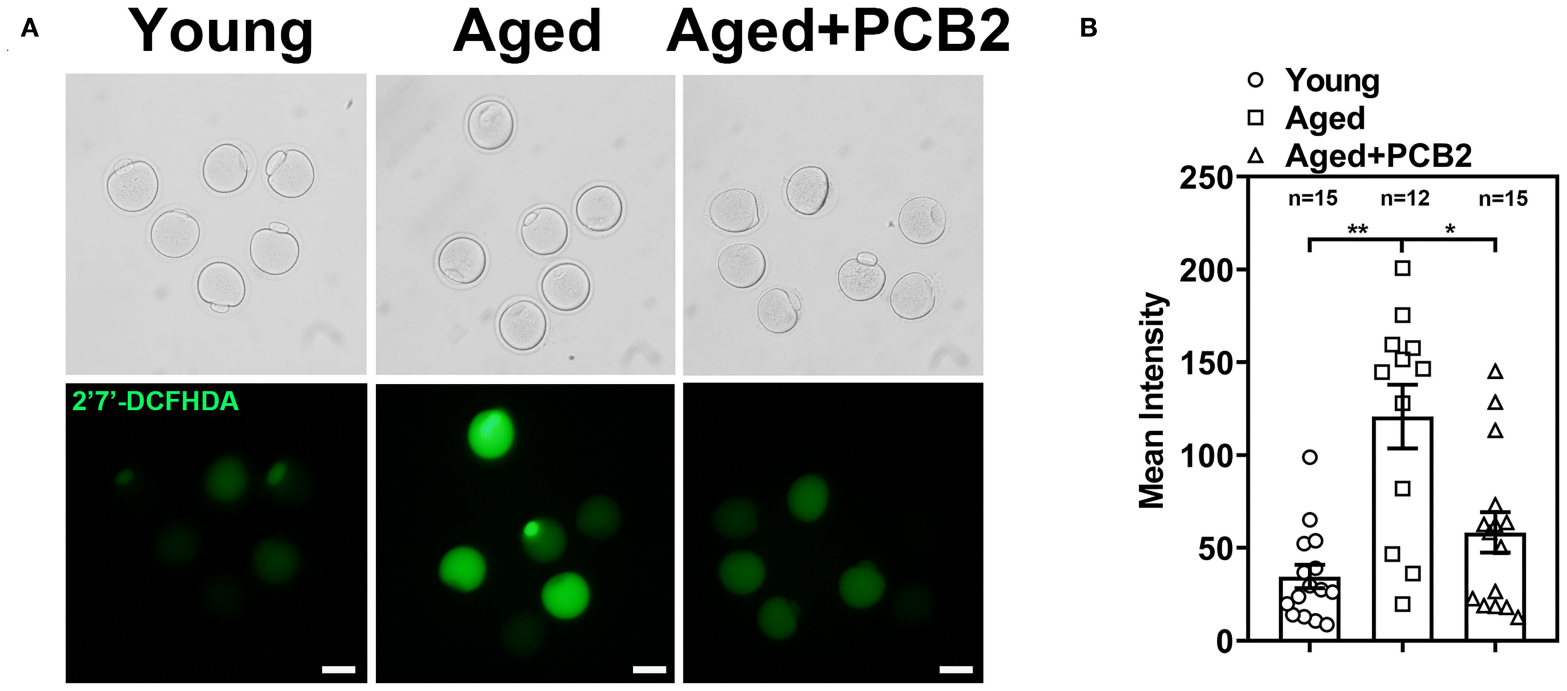
Effect of PCB2 supplementation on oxidative stress levels in aged oocytes. **(A)** Representative images of ROS levels in different groups. Scale bar, 50 μm. **(B)** Quantification of ROS fluorescence intensity was recorded in different groups. Data are presented as mean percentage (mean ± SEM) of at least three independent experiments. *P < 0.05, **P < 0.01.

Our previous study indicated that reproductive aging can impair mitochondrial function and further induce increased ROS levels in oocytes (16). Therefore, we further investigated whether PCB2 treatment could alleviate the oxidative stress in aged oocytes. We used 2′7′-DCFHDA to measure the intracellular ROS level (Figure 5A). PCB2 treatment significantly decreased the ROS level of aged oocyte caused by reproductive aging (young: 34.59 ± 6.33, *n* = 15; aged: 120.76 ± 17.16, *n* = 12; aged+PCB2: 58.33 ± 10.92 pixels, *n* = 15, *P* < 0.05) (Figure 5B).

## Discussion

Decreased oocyte quality is one of the irreversible damages due to aging (17). Oocyte quality can be influenced by reproductive aging through nuclear and cytoplasmic maturation during oocyte development (18). In addition to the nuclear and cytoplasmic dynamics, other changes occurred in the cortex of the aged oocyte also attribute to the declined developmental capacity (19). It was reported that abnormal distribution and decreased amount of cortical granules (CGs), which are golgi apparatus-derived vesicles that localize under the oocyte subcortex, were induced by reproductive aging in mouse oocytes (20).

Exogenous antioxidants can alleviate the decrease in oocyte quality caused by reproductive aging (8, 20). Procyanidins are a class of natural plant polyphenols, which have strong antioxidant properties (11, 21). We previously found that 5 μg/mLPCB2 supplementation during *in vitro* maturation progress could attenuate meiosis defects, improve the subsequent developmental potential after parthenogenetic activation of diabetic mouse oocytes (12). In agreement with our previous findings, the GVBD rate was significantly decreased in the aged oocytes, while the PBE rate was similar (16). As we pointed out in our previous report, aged oocytes underwent both GVBD and PBE more slowly than young oocytes, this indicated that the meiotic process in the aged oocytes was disrupted to a certain extent (16). In the present study, we found PCB2 supplementation can significantly improve the GVBD rate in aged oocytes (Figure 1). As the most numerous organelles in the cytoplasm, mitochondria supply the ATP needed for the oocyte to support critical events including maturation and spindle formation (22, 23). Previous study showed that improved mitochondria function alleviated meiotic defects (20). Our research indicated that PCB2 could improve the viability and restore mitochondrial function of vitrified-thawed oocytes (unpublished data). The present finding proved that PCB2 played a pivotal role in oocytes meiosis resumption, suggested that PCB2 might also contribute to maintain mitochondrial function in aged oocytes under oxidative stress.

Apart from the antioxidant property, we also discovered that PCB2 served as a cortical tension enhancer in aged oocytes. It has been reported that the aged oocytes are related to the changes in actin cytoskeleton integrity, loss of dynamic activity, and clump formation in actin (24, 25). The polarity of the cortex is mediated by the polarization of the oocyte, including the translocation of the spindle to the cortex in an actin-dependent manner, the enrichment of microfilaments to form the actin cap, the redistribution of cortical granules (CGs) to form a CGs-free domain, and microvilli loss in the region overlying the spindle (26, 27). During reproductive aging, aged mouse oocytes exhibited cortical polarity degradation (28). Similarly, aberrant cortical mechanics and actomyosin cytoskeleton functions were also found in post-ovulatory aged oocytes (7). Actin, myosin-II, and the ERM (Ezrin/Radixin/Moesin) family of proteins are enriched in complementary cortical domains and mediate cellular mechanics in mammalian oocytes (5). ERM is active through its phosphorylated form (pERM) and myosin-II contractility works with actin to mediate cortical tension and is regulated by phosphorylation of the myosin-II regulatory light chain (pMRLC) (7). In the present study, we found decreased cortical tension in reproductive aged oocytes. pERM activity was significantly decreased in cortical area in reproductive aged oocytes, which was consistent with post-ovulatory aged oocytes. By using a cVCA construct to decrease cortical tension in mouse oocytes, the newly established extra-soft mouse oocytes show impaired chromosome alignment, mainly due to a cytoplasmic increase in myosin-II activity (29). This was also consistent with our findings that the distribution of cytoplasmic pMRLC increased sharply and cortical pMRLC decreased significantly in aged oocytes (Figure 3). In post-ovulatory aged oocytes, anti-pMRLC signals were reduced in amicrovillar localization (subcortical spindle region), and showed aberrant amicrovillar morphology, such as a protruding amicrovillar domain, uneven pMRLC distribution around the boundary of the amicrovillar domain, and patchy pMRLC over the amicrovillar domain (7). But in our findings, reproductive aged oocytes mostly exhibited reduced pMRLC signals in amicrovillar domain, and clustered distribution in the cytoplasm (Figure 3A). The results demonstrated that the cortical tension was altered during aging process, and the discrepancies in pMRLC distribution pattern implied that different mechanisms underlying reproductive and post-ovulatory aging. This phenomenon was one of the abnormal distribution of post-ovulatory aged oocytes. Through immunofluorescence co-staining, we found that aberrant spindle formation and abnormal chromosome alignment were also increased in aged oocytes (Figure 4). Cheng et al. have pointed out that aged oocytes underwent GVBD and PBE with similar efficiencies and kinetics as young oocytes, but aging can cause an increase in chromosome aneuploidy of MII oocytes (30). Indeed, our results also suggest that aging induced meiosis defect does not necessarily affect the rate of the PBE. Cohesion forces on the centromere and hold the sister chromatids together (31). However, the activity of cohesion decreases with age and makes recombinant chromosomes prone to mis-segregation (2). Reports also indicated that intracellular pH (pHi) was elevated in aged oocytes, the elevated oocyte pHi might be related to the loss of cohesion and the increased aneuploidy in aged mouse (30). This indicated that aging can induce abnormal chromosome alignment and eventually lead to aneuploidy. However, the extrusion of first polar body is a dynamic process, variations in the PBE rate is largely dependent on the observation time points. Although the proportion of PBE was similar to that of young oocytes, the increased proportion of chromosome misalignment in old oocytes suggested that there was a defect in the monitoring mechanism during chromosome separation. This may explain our results that reproductive aging induces decreased cortical tension, increased cytoplasmic myosin-II activity, and further disrupts normal spindle morphology and chromosome alignment. These results are consistent with the findings in post-ovulatory aged oocytes, both reveal the relevance of cortical tension to normal oocyte function (5, 7). Furthermore, PCB2 supplementation restores decreased expression of pERM and pMRLC, alleviates abnormal spindle formation as well as chromosome misalignment. It was reported that cell mechanics changes affected the shape and function of the mitotic spindle (32). Our result also indicated that spindles exhibited morphological differences in response to cortical tension variations.

Reproductive aging not only impair oocytes meiosis resumption, but also affect the mitochondrial Ca^2+^ homeostasis and mitochondria function (16). The proper distribution of mitochondria plays a key role in the regulation of redox homeostasis and is necessary for cell survival (33, 34). We previously demonstrated that maternal age could disturb mitochondrial distribution, accompanied by overload mitochondrial Ca^2+^ level and decreased mitochondrial heat production (16). Alterations in mitochondrial function permit aging cells to regulate senescence phenotypes induced by DNA damage, which include the overproduction of ROS via mitochondrial dysfunction (34, 35). Since the robust interplay between increased ROS level and mitochondrial dysfunction, the observed reduced ROS level after PCB2 treatment proved our assumption that PCB2 could improve mitochondrial function in aged oocytes. In the present study, we found that the decreased cortical tension and increased ROS level in oocytes from maternally aged mice was associated with meiotic defects, which was consistent with previous findings that increased ROS level and reduced cortical tension in post-ovulatory aged oocytes further induce oocyte cortex and spindle abnormalities (3). To divide, cells dramatically change shape and round up against extracellular confinement (36). It was discovered that the mechanical characteristic of extracellular matrix could be perceived and its variation was associated with mitochondrial function alterations (34). In the present study, abnormal spindle formation and chromosome arrangement induced by cortical tension and oxidative stress in aged oocytes were corrected by PCB2, probably attributed to the ameliorated mitochondrial function. Other antioxidants such as melatonin, resveratrol and mogroside V, have been reported to improve oocytes quality through ameliorating mitochondrial function, but the effect of antioxidants on cortical tension has not been reported. Our results implied that there was an intricate relationship between mitochondrial function and cortical tension in aged oocytes. To our knowledge, this is the first report identified the additional role of PCB2 played in cortical tension regulation in aged oocytes.

## Conclusion

In conclusion, our results indicated that PCB2 could protect reproductive aged oocytes from meiotic abnormalities by increasing cortical tension, which correlates with restored redox homeostasis under maternal age-induced oxidative stress. Overall, our work provides insights into the mechanism underlying age-related increase in oocyte meiotic defects, and expounds the theoretical basis for application of PCB2 to improve oocyte quality in aged women.

## Data Availability Statement

The original contributions presented in the study are included in the article/supplementary material, further inquiries can be directed to the corresponding author.

## Ethics Statement

The animal study was reviewed and approved by Institutional Animal Care and Use Committee of China Agricultural University.

## Author Contributions

QZ, JL, and XF conceived and designed the study. QZ, GZ, XD, and HL performed experiments, collected data, and analyzed data. QZ, XF, and JL wrote the initial manuscript. YH and PW revised the manuscript. All authors have read and agreed to the published version of the manuscript.

## Funding

This work was funded by Chinese Universities Scientific Fund, Grant/Award Number: 2021TC061; Natural Science Foundation of Hebei Province, Grant/Award Number: H2020206254; Special Program for Training and Guiding Outstanding Young and Middle-Aged Talents, Grant/Award Number: SKLSGIHP2021A01; National Natural Science Foundation of China, Grant/Award Number: 81901562 and 31372307; Key Research and Development Projects in Hebei Province, Grant/Award Number: 18226604D; Xinghuo Program of the First Hospital of Hebei Medical University, Grant/Award Number: XH202005.

## Conflict of Interest

The authors declare that the research was conducted in the absence of any commercial or financial relationships that could be construed as a potential conflict of interest.

## Publisher's Note

All claims expressed in this article are solely those of the authors and do not necessarily represent those of their affiliated organizations, or those of the publisher, the editors and the reviewers. Any product that may be evaluated in this article, or claim that may be made by its manufacturer, is not guaranteed or endorsed by the publisher.
